# A hypervascular placental polyp after complete abortion: a case report

**DOI:** 10.1186/s12905-023-02672-x

**Published:** 2023-10-10

**Authors:** Ryan Spielvogel

**Affiliations:** Department of Family Medicine, Sutter Medical Group, 1201 Alhambra Blvd. Ste 300, Sacramento, CA 95816 USA

**Keywords:** Uterine bleeding, Retained products of conception, Puerperal disorder, Case report, Placental polyp, Abortion

## Abstract

**Background:**

Placental polyps are rare complications of delivery or abortion. They are thought to complicate less than 0.25% of all pregnancies, although the actual incidence is unknown. While they typically occur within four weeks of delivery or abortion, they can have a variable presentation, which can lead to a delay in care.

**Case presentation:**

A 35-year-old G4P2012 patient presented at 9 weeks gestation for a medication abortion. Post-abortion ultrasound after one week confirmed the abortion was complete and her bleeding ceased. The patient then presented two months later with the new onset of worrisome bleeding. She was found on ultrasound to have a new hypervascular polypoidal mass in the endometrial cavity. She then underwent an in-office dilation and curettage with an electric vacuum aspirator, which was curative. A follow up ultrasound three months later demonstrated no recurrence.

**Conclusions:**

Placental polyps are a rare complication following pregnancy and should be included in the differential when a patient presents with bleeding and a new mass in the endometrial cavity on ultrasound following a delivery or abortion, even when frankly retained products of conception had been ruled out at time of abortion.

## Background

Placental polyps are polypoidal masses composed of degenerating, fibrotic chorionic villi found within the endometrial cavity. Although the exact mechanism of pathogenesis is unknown, placental polyps are thought to occur after delivery or abortion when a retained fragment of chorionic tissue in the endometrial cavity forms into a polypoidal mass [1]. While doppler ultrasound and the patient’s history can aid in diagnosis, definitive diagnosis is made histopathologically [2]. Placental polyps occur in fewer than 0.25% of pregnancies, with only 6% of those being hypervascular and associated with severe hemorrhage [2, 3]. While most cases present within four weeks of parturition or abortion, cases have been reported months or even years later [4]. Human chorionic gonadotropin (hCG) levels are frequently positive.

As reported in the literature, typical cases of placental polyps present with persistent bleeding following parturition or abortion [2]. Management is via hysteroscopic resection or dilation and curettage. Here we present the case of a hypervascular placental polyp after a documented complete abortion and who initially had cessation of bleeding.

## Case presentation

A 35-year-old patient, G4P2012, presented to our outpatient clinic in Sacramento, California, at 9 weeks 0 days gestation by last menstrual period, confirmed by two-dimensional (2D) transvaginal ultrasound that day, for a medication abortion. Other than a single prior miscarriage, she had no prior history of pregnancy complications including hemorrhage or cesarean section. Medical and surgical history were otherwise noncontributory.

**Fig. 1:**
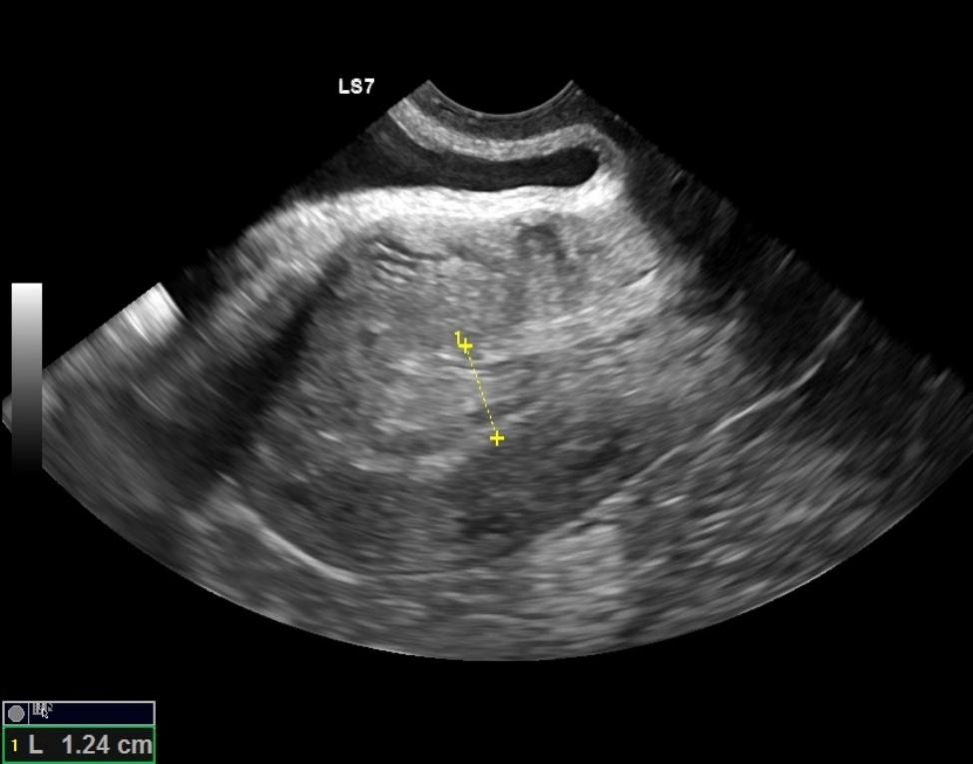
Transvaginal sagittal 2D ultrasound image one week after medication abortion. Ultrasound revealed expected thickened endometrial complex and absence of gestational sac, which was consistent with a medication abortion that was complete. No obvious retained products of conception were present

Patient underwent a medication abortion that day with mifepristone 200 mcg and misoprostol 800 mcg. She reported heavy bleeding for the following day, which then slowed down considerably. Follow up office-based 2D transvaginal ultrasound one week later revealed a medication abortion, which was complete with a thickened endometrial complex, but no obvious retained products of conception (Fig. 1).

**Fig. 2:**
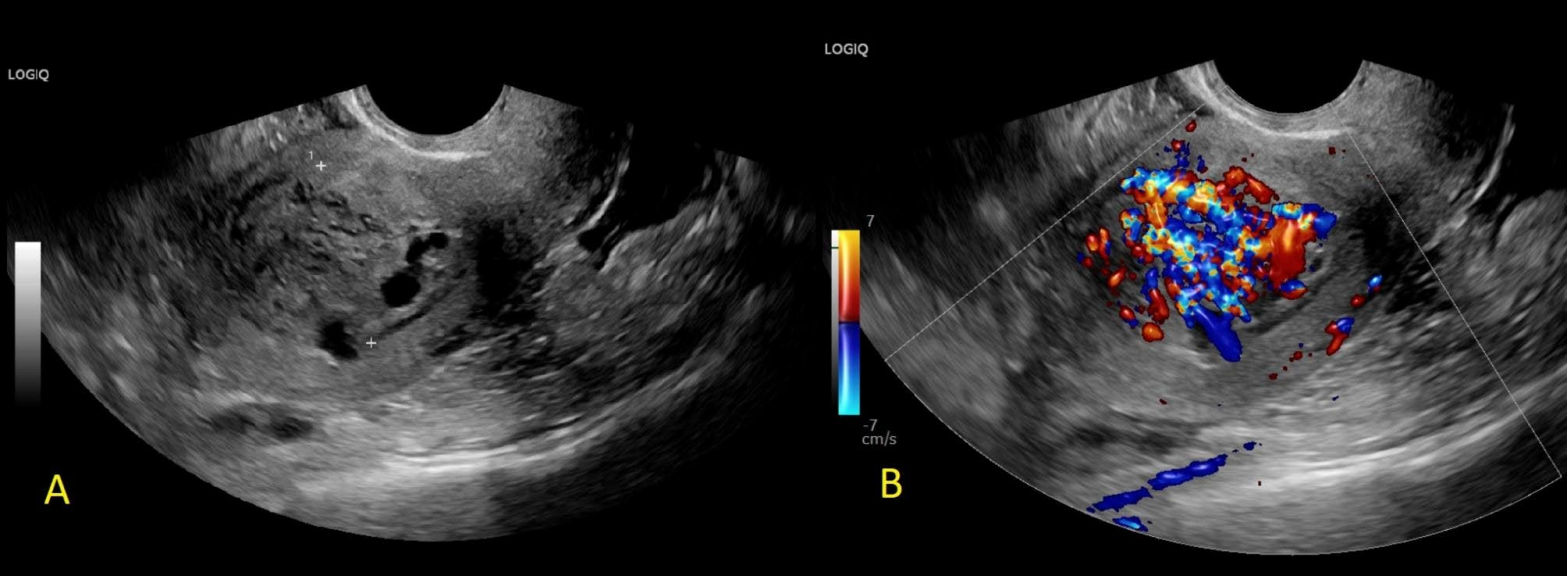
Transvaginal sagittal 2D ultrasound (**A**) with color flow doppler (**B**) demonstrating a new mass in the endometrial cavity on presentation to the outside emergency department eight weeks after medication abortion. Comment from radiology was, “Markedly thickened endometrium with heterogeneous appearance and marked vascularity.”

Bleeding stopped shortly thereafter and then restarted six weeks later. After five days of heavy bleeding, she presented to the emergency department of a different medical institution where she was found to have a normal hemoglobin (12.0 g/dL) and negative quantitative hCG. 2D Transvaginal ultrasound revealed a new hypervascular mass in the endometrial cavity measuring 3 cm (Fig. 2). Because the hemoglobin was within normal limits, the patient was discharged home with 800 mcg of misoprostol to take orally and was instructed to follow up with her outpatient physician for further management.

**Fig. 3:**
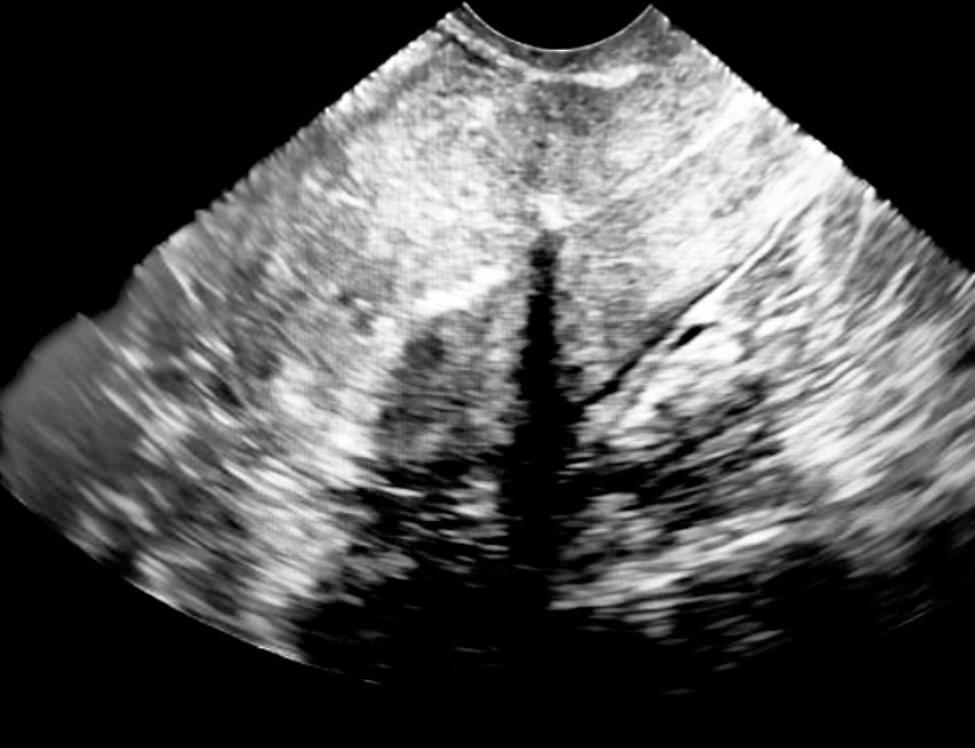
Transvaginal sagittal 2D ultrasound after evacuation of mass in the endometrial cavity by dilation and curettage. Ultrasound demonstrates resolution of the mass in the endometrial cavity

In the days following misoprostol use, the patient reported passage of clots without tissue, and the bleeding persisted. Five days following the emergency department encounter, the patient was seen again in our office. 2D transvaginal ultrasound redemonstrated the large hypervascular mass in the endometrial cavity, at which point a placental polyp was suspected. In two passes, the mass was evacuated with electric vacuum aspiration with post-procedure imaging demonstrating an empty uterus (Fig. 3). Post-procedure bleeding was minimal, and the patient was discharged home without further events.

The specimen was sent to pathology, which showed “degenerating and inflamed decidua and fibrinous debris. A few degenerating chorionic villi are also noted.” This confirmed the suspected diagnosis of placental polyp. The patient’s normal menstrual cycles resumed the following month. Three months later, a repeat office-based 2D transvaginal ultrasound showed a continued thin endometrial stripe of the uterus and confirmed the full resolution of the placental polypoid mass.

## Discussion and conclusions

We present the case of a hypervascular placental polyp after a medication abortion, which was ultrasound-confirmed to be complete. Owing to their relative rarity, the pathophysiology of placental polyps is poorly understood. They are thought to arise from a small amount of retained products of conception that undergoes neo-vascularization over time [5]. Placental polyps typically present with persistent bleeding after delivery or post-abortion. While they are most frequently associated with a persistently low level of positive serum hCG, they can also present with a negative serum hCG as in our case [3–5]. Various treatments have been reported, most commonly with dilation and curettage or hysteroscopic resection [2–4]. However, expectant management, use of gonadotropin releasing hormone antagonists, and transarterial embolization have also been reported [6, 7].

Acquired uterine arteriovenous malformations (AVMs) can have similar clinical characteristics to placental polyps. Ultrasonography and or Computed Tomography Angiography (CTA) can generally distinguish between the two as AVMs form within the myometrium rather than the endometrium [3]. Diagnosis can be challenging though if the AVM directly abuts the endometrial cavity. AVMs are static lesions, however, compared to placental polyps, which develop over time. In our case, AVM was ruled out since the lesion was not present at the time of the ultrasound confirming abortion completion and it developed between then and her later presentation. Proper diagnosis of a placental polyp is paramount prior to treatment as attempted dilation and curettage on an AVM could result in life-threatening hemorrhage. Endometrial polyps also present as masses in the endometrial cavity and can be distinguished from placental polyps by the presence on doppler ultrasound of a feeding artery, known as pedicle artery sign [8].

Our case was unusual in several respects. Most cases reported in the literature have their first imaging of the polyp documented at the time of presentation. Our case has the benefit of a documented post-abortion 2D transvaginal ultrasound image showing only a thickened endometrial stripe and no frankly retained products of conception consistent with a medication abortion, which was complete. Additionally, most cases reported in the literature describe persistent bleeding as the presenting symptom, which prompts patients to present within days to several weeks of delivery or abortion. However, our patient had full cessation of bleeding after her medication abortion, which was again consistent with a medication abortion that was complete at the time.

This case illustrates and supports the speculation that placental polypoid masses represent a proliferation and neo-vascularization of a subclinical amount of retained chorionic tissue and are not simply a static section of the frankly retained products of conception. Moreover, this case illustrates that the amount of chorionic tissue needed to form such a mass can be quite small since it evaded detection on the initial post-abortion 2D transvaginal ultrasound and presumably needed time to grow before bleeding restarted.

In conclusion, this case demonstrates an unusual presentation of a hypervascular placental polyp after 2D transvaginal ultrasound imaging showed a medication abortion that was complete. Physicians taking care of pregnancy-capable individuals should keep placental polyps on the differential when a patient presents with delayed postpartum or post-abortion bleeding, even when frankly retained products of conception were previously ruled out. Written informed consent to publish was obtained from the subject of this case presentation.

## Data Availability

Data analyzed during current study is available from corresponding author upon reasonable request.

## List of abbreviations

2D: Two-dimensional
AVM: Arteriovenous malformation
CTA: Computed Tomography Angiography
hCG: Human chorionic gonadotropin
